# The current state of forensic imaging – post mortem imaging

**DOI:** 10.1007/s00414-025-03461-x

**Published:** 2025-03-24

**Authors:** Fabrice Dedouit, Mathilde Ducloyer, Jamie Elifritz, Natalie L. Adolphi, Grace Wong Yi-Li, Summer Decker, Jonathan Ford, Yanko Kolev, Michael Thali

**Affiliations:** 1 Department of Forensic Pathology, Bâtiment Raymonde Fournet, Place du Dr Baylac, Hôpital Purpan, Toulouse, 31700 France; 2 Department of Forensic Pathology, Nantes University, University Hospital, Bd Jean Monnet, Nantes, F- 44000 France; 3 Forensic Radiology Group, Anderson, SC USA; 4 Office of the Medical Investigator, University of New Mexico, Albuquerque, NM 87131 USA; 5 Department of Radiology, Penang General Hospital, Jalan Residensi, Georgetown, Penang 10450 Malaysia; 6 Departments of Radiology and Pathology, University of Southern California Keck School of Medicine, 1450 San Pablo Street, Suite 3500, Los Angeles, CA 90033 USA; 7 Department of General Medicine, Forensic Medicine and Deontology, Medical University - Pleven, 1 St Kliment Ohridski str., Pleven, 5800 Bulgaria; 8 University Zurich, Virtopsy Group, Switzerland

## Abstract

Over the last few decades, forensic imaging has become an essential part of current forensic practice. The aim of this 4-part review is to provide a comprehensive overview of forensic imaging over the first 25 years of this century. After a brief historic review, this first part details the advantages and limitations of post-mortem imaging for the indications most frequently encountered in daily practice.

## Introduction

Of all medical advances in recent decades, imaging has revolutionized medical practices profoundly. For the first time, we could see inside bodies without having to open them. Imaging has enabled us to make definite diagnoses, improve our understanding of the pathophysiological mechanisms of disease, propose screening methods and improve the diagnosis and treatment of cancers. On the technical front, considerable progress has been made since the first X-ray of the hand of Berta Roentgen. These advances include the invention of computed tomography (CT), magnetic resonance imaging (MRI) and ultrasound (US), the use of radiology to develop new treatment modalities with interventional imaging or digital imaging with picture archiving and communication systems. But beneath these technologies, imaging has profoundly changed our approach to the living or dead body. In forensic medicine, this has led to questioning the need to perform autopsies, accentuated by societal changes, that show that families and healthcare professionals are less and less inclined to this practice [1, 2]. The transition to the year 2000 was accompanied by the appearance of the term ‘virtual autopsy’, the scope and practical implications of which have since been clarified and qualified [3–5].

Depending on the country in which the forensic doctor works, he or she may perform forensic autopsies exclusively, or also be involved in clinical forensic medicine. This is also a part of the forensic imaging. This implies large possibilities of medicolegal diagnostics that can be included in forensic reports.

The word ‘autopsy’ is of ancient Greek origin and is a combination of ‘autos’ (self) and ‘opsomei’ (to see). It can be translated as “to see oneself” [6, 7]. Medico-legal autopsy has many objectives: to reconstruct the sequence of events and circumstances that preceded and led to death, including pre-mortem diseases, to determine the cause of death, its legal classification (homicide, suicide, accident or natural death), to establish the time of death and to identify the deceased [7]. In general, forensic imaging does not replace forensic autopsy and functions as a complementary tool for the forensic doctor or the forensic pathologist, even if some ideas of imaging as a “triage” tool have been described.

The first part of this review is to provide an update of postmortem imaging, without forgetting past important elements.

## A brief historic review

Radiography was integrated into the field of the forensic medicine and anthropology soon after the first radiographs (X-ray) was performed by Wilhelm Conrad Roentgen in 1895.

In fact, the usefulness of X-rays in revealing the hidden secrets of the body began to be put to practical use in the United States in 1895, and then in England in 1896 [8]. These first forensic radiographs were used for clinical and post-mortem purposes. Very early on, the possibility of locating bullet fragments on radiographs was emphasized. As early as 1896, X-rays were used by Koenig in Frankfurt on human and feline mummies and by practitioners in the Netherlands and Liverpool on ornithological mummies [9]. Without unpacking the mummies, Koenig was able to distinguish between mature and immature and human and non-human subjects. The interaction between radiological imaging and paleopathology, anthropology and archaeoanthropology is called paleoradiology [9].

These are important facts and steps, because they perfectly illustrate the early interaction between two different medical specialities: *radiology* and *forensic pathology*. Interestingly, things are not so different today: radiology can help legal medicine in clinical forensic medicine and in forensic pathology. These first X-rays in 1896 in forensic and anthropological fields were followed by hundreds of publications at the end of the XXth and the beginning of the XXIst centuries.

Following the same trend, the realization of CT in forensic and archeological contexts was performed very early after the development of CT by Hounsfield and Cormack in the early 1970s. The first CT in archaeological context was completed in 1977 on the brain of an Egyptian mummified child [10]. The same year, Wullenweber performed the first forensic CT in a context of ballistic trauma [11]. The forensic use of MRI began in 1986, on a mummified ankle after a complex protocol of rehydration [12]. Previous attempts of scanning mummies using MRI failed, but Rühli succeeded by using specific short TE sequences, without rehydration of the remains [13].

A major milestone was written by Prof Byron Gil Brogdon, with whom the real history of modern forensic imaging began. The book “Forensic Radiology” was one of the first pillars of this new discipline [8]. Since 1998, of course, many books, manuals and articles have been written illustrating the interaction between radiology and forensic medicine. This has allowed potential links and collaborations to be highlighted. In recent years, it was Prof Richard Dirnhofer from the Forensic Institute in Bern (Switzerland) who, together with Prof Peter Vock from the Department of Radiology, founded the “Virtopsy Project” in 1998 [4]. Like all successful ventures, this was a human and a scientific venture. This project allowed the scientific community to realize that modern imaging has an amazing potential for forensic medicine and pathology, just as it had and still has for clinical medicine and surgery. The dissemination of this work, and of course of other research groups, contributed to the creation of the new medical discipline of forensic radiology.

Some specific working groups were successively created, in order to promote forensic imaging. We can cite the “Technical Working Group Post-mortem Angiography Methods” (TWGPAM, which focuses on post-mortem injection of contrast agents), the AGFAD (“Arbeitsgemeinschaft für Forensische Altersdiagnostik”, i.e. working group for forensic age estimation of the German Society of Forensic Medicine) or scientific societies (such as the “International Society of Forensic Radiology and Imaging” (ISFRI), in addition to the traditional national radiology or forensic medicine societies [14–17]. The improvement of forensic imaging is also supported by its inclusion in official national programs for the professional training of radiologists, radiographers and forensic pathologists.

## Applications of post mortem imaging

**Fig. 1:**
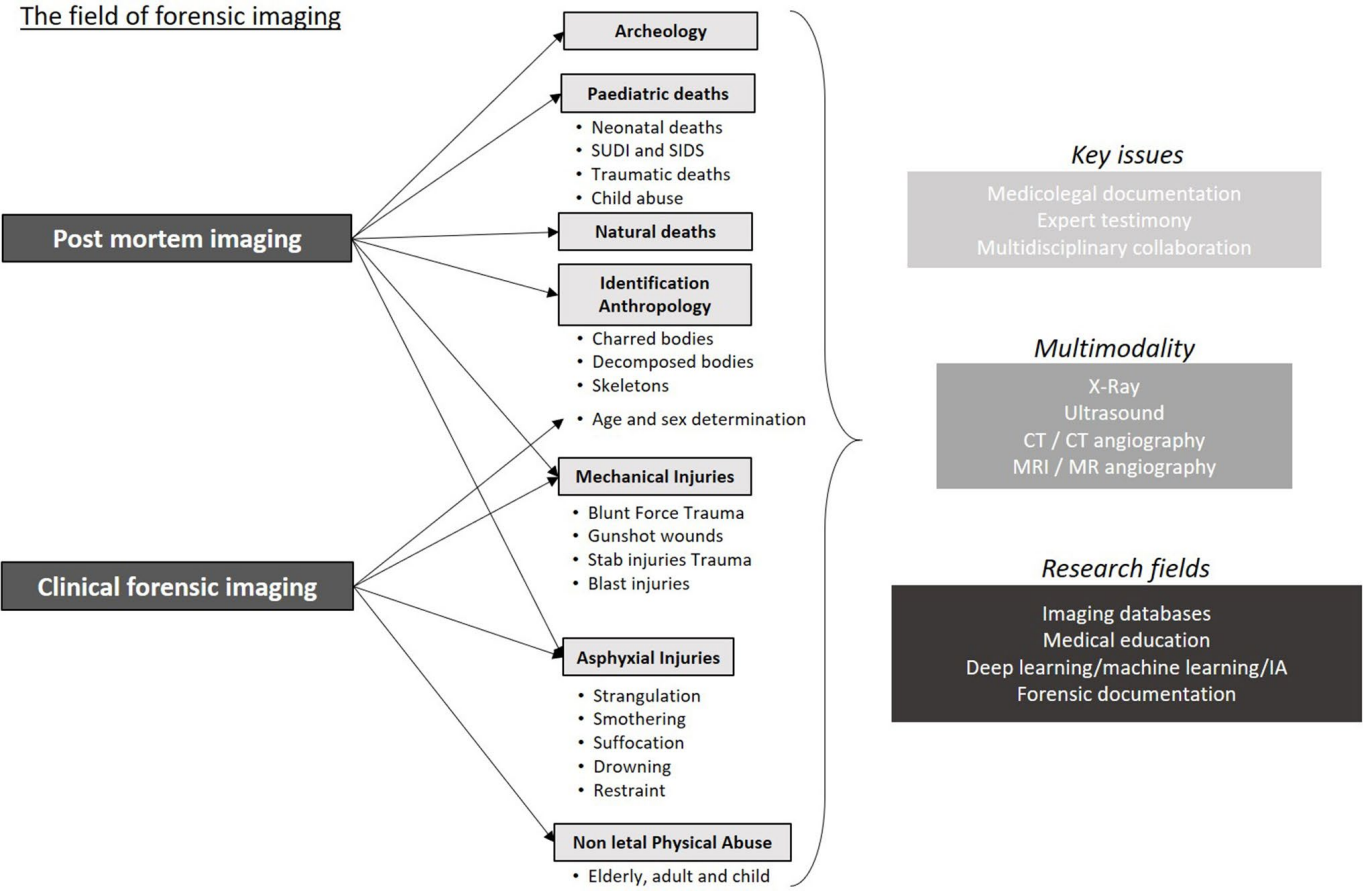
Illustration of the main indications and benefits of post-mortem imaging in the most common forensic situations

Although there is broad agreement within the medico-legal community about the benefits of forensic radiology, there are still many centres around the world that do not have access to modern imaging technologies. As a result, forensic radiology is still far from being a reality in many countries. Moreover, even when it is available, it must be used with care, and experts must be aware of the advantages and limitations of the radiological tools they use. The following paragraphs summarize the main indications and benefits of post-mortem imaging in the most common forensic situations. These indications are illustrated in Fig. 1.

### Gunshot wounds

Injuries secondary to gunshot trauma were the very first to be explored by imaging for forensic purposes [18]. The high sensitivity of X-rays to metallic material makes them an ideal tool for detecting projectiles, using fluoroscopy or CT scans. Historically, bullet identification was performed by radiography. Fluoroscopy was used in some instances to visualize foreign bodies in real-time, assisting retrieval.

PMCT offers many advantages over these modalities, as it not only can pinpoint the location of projectiles, but also provides information on their nature (single bullet, pellets or fragmented foreign bodies), add precision on the intra-body trajectory and helps the forensic pathologist to list the resulting internal injuries [19]. PMCT is the most precise imaging tool for triangulation of bullet location for retrieval. Many studies suggest the use of PMCT should be standard for all gunshot injuries [19–27]. Indeed, PMCT had superior results compared to CA for establishing trajectories and identifying injuries but the detection of entry or exit wounds by postmortem imaging remains more limited [28–30]. Some authors are proponents for PMCT and external examination in lieu of autopsy in select situations [25, 27, 31]. PMCT is especially helpful in cases where a bullet may not be expected [32, 33].

In addition to defining ballistic characteristics (trajectory, entry and exit sites, internal injuries), PMCT can specify associated or indirect lesions such as gas embolism or blood aspiration, which is highly reliable for establishing if a gunshot injury was ante- or postmortem [19, 34–37]. Application of metal artifact reduction, such as iterative reconstruction or single-energy metal artifact reduction, is a useful and simple technique that facilitates the description of intracranial injuries [38].

Typically, skeletal entry wounds demonstrate internal bevelling and exit wounds demonstrate external bevelling. Pelvic fractures, which may be concealed at CA are clearly depicted on PMCT. Bone fragments, gas, blood, and metallic fragments are displaced along the track of the wound, thus, evidencing bullet trajectory. Soft tissue disruption is most frequently evident by gas from cavitation effects. The gold standard for identification of abnormal gas in body cavities such as pneumocephalus, pneumorrhachis, pneumothorax, and pneumoperitoneum is PMCT.

The combination of abnormal gas, haemorrhage, and contour irregularities is highly suspicious for organ injury in the setting of gunshot wounds. Depending on the number and sequence of additional injuries, haemorrhage may not be present. When present, haemorrhage may be challenging to identify (especially in the regions of the liver and spleen where blood is isoattenuating to the organ). In some instances, regional haemorrhage may be a red-herring, related to a separate injury. As with blunt force injuries, PMCTA can enhance the depiction of solid organ injury and identify the precise locations of vascular injuries [15, 20, 39]. In fact, based on the evidence, Minoiu et al. recommend that PMCTA should be incorporated into standard practice in forensic investigations of gunshot wounds [40]. PMMR can also provide additional information on intracranial bleeding and could help to understand the lesional mechanisms of permanent and temporary cavitations [41–44].

Last, critical elements of injury reconstruction are aided by the reliability and reproducibility of measurements on PMCT [26]. PMCT images can be further implemented with 3D processing, allowing sterile images for court. These demonstrative aids are powerful in depicting injuries to laypeople [21, 45].

### Blunt force injuries

The body of evidence supporting imaging in the context of blunt force injury is irrefutably vast and conclusive. Like clinical and medical imaging, PMCT (without and with contrast) is the preeminent modality for evaluation of traumatic injuries. Numerous studies have demonstrated the utility of PMCT in the context of blunt force injury (i.e. fatal traffic and train accidents, fall from height, intentional violence (with or without blunt objects), domestic accidents) [46, 47].

On comprehensive analysis, Donchin et al. reported in 1994 that PMCT revealed 70.5% and autopsy identified 74.8% of “pathologic states” in trauma victims [46]. Since then, numerous publications have echoed the reliability and reproducibility of PMCT in medicolegal forensic investigations. Sifaoui et al. found that noncontrasted PMCT had a sensitivity of 21% when confirming the injury with direct signs on imaging. However, sensitivity increased to 74% using indirect signs [48]. Overall, it was reported that noncontrasted PMCT has a sensitivity of 71% for detecting all major injuries. Perhaps the most profound anatomical location to capitalize on PMCT for skeletal injury is the cervical spine. Cross sectional images readily demonstrate subtle injuries that may be unexpected and are difficult and/or not commonly dissected at autopsy [49, 50].

A systematic review by Jalalzadeh et al. further validated PMCT, determining that while CA is the gold standard for the detection of organ and soft tissue injuries, the primary and most important secondary injuries related to the cause of death are accurately visualized by PMCT [51].

One of the most powerful prospective studies championing the value of PMCT in blunt force injury is Lathrop et al.’s recent publication. In this article, the sensitivities for identifying blunt force injury in PMCT is 74% and 73.1% for autopsy [52]. Even more compelling, is the capacity of PMCT to accurately identify injuries required for certifying cause of death. Lathrop’s study found that the cause of death assigned from the PMCT interpretation was correct 88% of the time versus 95.8% for autopsy. Since most radiologists have limited experience in assigning cause of death, it is logical to assume that their accuracy could improve with experience and education. Likewise, forensic pathologists with imaging experience would be able to more frequently assign the correct cause of death using PMCT.

As previously reported, PMCT is superior to conventional autopsy for identification of skeletal injuries and for detection of pathologic gas collections. PMCT performs less well for the identification of superficial soft tissue lesions (which are easily identified at external examination) and for the diagnosis of some internal soft tissue injuries. The shortcomings of PMCT for evaluation of soft tissue injuries are abated by PMCTA [39, 53–58]. A multicenter study by Grabherr et al. determined that if autopsy had been performed without PMCT and PMCTA that 39% of findings and 23% of essential findings would not have been reported, and consequently, the autopsy report of some cases would be wrong. It was reported that PMCTA had a particular value over autopsy in identification of vessels of small caliber or those located in difficult to access anatomical locations [39]. Correspondingly, other studies found that PMCTA has a higher sensitivity of identifying the exact arterial or venous vessel injured compared to CA [53, 59, 60]. Like PMCT, PMCTA provides a permanent uncorrupted record of injury open to audit at any timepoint.

### Sharp force

Evaluation of sharp force injuries with imaging is best aided by PMCT and further enhanced by PMCTA. Pathologic tracks or collections of gas are the predominant indication of sharp force injuries on PMCT. Hemorrhage, soft tissue injuries (including organ damage), and skeletal injuries may coexist. Correspondingly, PMCT is superior to conventional autopsy for diagnosing pneumothorax, pneumomediastinum, mediastinal shift, and pneumoperitoneum [61, 62]. In the absence of gas (which could be related to body positioning) fat infiltration may be seen along the path of the weapon [22]. A fortunate forensic pathologist may even visualize the tip of a broken weapon residing in the body [63].

While PMCT is insensitive for superficial wounds (especially in the absence of skin reconstructions) injuries responsible for the cause of death are generally well-depicted. In fact, Schnider et al. found that 87.3% of deep and potentially lethal stab wounds were identified by PMCT [64]. PMCT aids autopsy planning and reveals all skeletal injuries, providing vital information that can be utilized for weapon identification [64, 65].

PMCT lacks the sensitivity to differentiate between arterial and venous wounds. Multiplicity of large grouped wounds also may not be acknowledged, likely because the proximity of many grouped lesions obscures proper identification. PMCTA may detect subtle injuries to small vessels that may not be routinely dissected at CA [66, 67]. In addition to identifying lethal injuries, PMCT and PMCTA can be used to reconstruct crucial evidence such as weapon directionality, provide demonstrative aids for educational and courtroom proceedings, and even be used as a tool for weapon identification (with the incorporation of advanced technologies such as 3D printing) [67, 68].

### Drowning

The forensic evidence of drowning that can be described by the forensic pathologist during the external and internal examination of a body found in water remains mostly non-specific and inconstant [69]. The use of PMCT provides convincing evidence in favour of drowning based on several signs: the presence of fluid in sinuses (maxillary, sphenoidal and frontal) and in the mastoid air cells. The presence of sediment in sinuses, trachea and bronchi, and stomach as well as diaphragm lowering are the most indicative imaging signs in favour of drowning [70–73]. Postmortem imaging also helps to detect pleural effusion, very frequently found in drowning cases [74]. The presence of sediment in the aero digestive tract in particular, is perhaps the most specific clue [73]. Other signs such as the presence of fluid in the trachea bronchial tree, haemodilution (estimated by the measurement of blood density in heart cavities), enlarged lungs and gastric distension have been described but are non-specific to drowning cases [72]. Last, post mortem imaging will provide crucial elements for differential diagnosis, by excluding traumatic injury and detecting foreign bodies, and for identification.

### Identification

Solving the question of the correct identification of an unknown body is an important part of forensic practice. Forensic imaging contributes to the identification process in several ways: by highlighting joint prostheses and osteosynthesis material, by detecting and describing dental care, by looking for rare anatomical variants and, last but not least, by enabling a comparison of ante- and post-mortem imaging, called radiological identification [75]. Imaging is the basis for pattern recognition but can also be used to exclude a previously presumed “match” or identification. Any imaging method or combination of imaging methods can be employed (X-ray, CT scan or even MRI), but it is undoubtedly the CT scan that remains the most reliable and available modality, generating volumetric data that can be reconstructed in different imaging planes, employed for maximum and minimum intensity projections (MIP, MinIP), constructed into three-dimensional renderings and printed three-dimensional models [76, 77]. In theory, any imaged anatomical finding can be utilized for comparison, keeping in mind that the greater the number of lines of evidence, the more distinctive the findings, and the stronger the multidisciplinary approach, the more robust the radiological identification process will be. Hatch et al. characterized the following categories for lines of evidence: normal anatomy, anatomic variants, disease (inclusive of natural disease, prior injury, and medical interventions), and dental [75]. Convincing results are obtained by comparing the shape of the paranasal sinuses [78–81]. Thick slab MIPs can be rendered for direct comparison to radiographs and curved MPRs can be compared to orthopantograms for an “apple to apple” comparison [75]. Comparison of the shape of the sternum is also possible [82].

The works of Mann and Kahana, Quatrehomme et al. confirmed more recently the usefulness of the comparison of bone trabeculae for positive identification [83–85]. The main constraint of this simple method is to obtain an incidence and a spatial resolution strictly identical to the ante mortem X-ray, which can be difficult in incompletely skeletonized bodies.

Although there are no national or international recommendations for radiologic identification, many publications and professional societies have addressed best practices relating to disaster victim identification [76, 86–93].

### Charred bodies

In addition to serving as a powerful tool of identification in cases of charred remains, PMCT provides critical internal information about these challenging cases. As previously mentioned, PMCT is an excellent instrument to identify foreign bodies, such as bullets, which are particularly difficult to identify and locate in carbonized remains and may be related to the cause of death. The presence of pulmonary oedema can signify the individual was alive at the beginning of the fire [94]. Sharp force wounds, in contradistinction to thermal injuries, will demonstrate clean edges with evidence of subcutaneous gas surrounding the cut lines [95].

PMCT can aid forensic investigations by recognizing fluid-fluid levels (an indication of non-coagulated blood) in the cardiac cavities and great vessels for targeted high-quality sampling [95]. Similarly, the presence or absence of fluid in the urinary bladder can be assessed to aid procurement. Another highlight of PMCT of charred remains is the ability to detect the presence of skeletal and intracranial injuries and to differentiate traumatic versus heat-related pathology. We can cite epidural collections with a subdural appearance typical of thermal injury, to be differentiated from ante mortem injuries [96, 97]. Thermal changes in the cranium present as fine fracture lines in the outer table followed by delamination of the outer table in multiple fragments with an intact inner table. Extremity fractures occur in exposed bones, initially presenting as fine linear fractures, followed by thermal amputation and the characteristic “flute mouthpiece” morphology [98]. Bone marrow lucencies may demonstrate a mottled pattern in regions of direct fire exposure, directing the investigation of the fire outbreak [95] (Table 1).

**Table 1:** Summary of the recommendations dealing with PMCT recommendations of the included National and international societies. N/A: Not applicable

Organization	Published Date	PMCT Applications	PMCT limitations	PMCT Technical Recommendations	Education and Training Recommendations	Other high yield topics
Swiss	2014	*Not specifically listed *Note that PMCT is a routine practice on most cases	*Not specifically listed	General recommendations	“Experts”	Radiation protection, preparation and transportation of the body, preparation of the body, Data archiving,
German Working Group	2015	*Written specifically for PMCT PMCT as a rule: *Homicide *Radiopaque foreign bodies *Child abuse/infanticide *Unexpected deaths in young children (up to 6 years) *Air/gas embolism PMCT case by case basis: *Accidental death *Suspected treatment errors Unexpected deaths up to age 17 years *Highly altered corpses *Unidentified corpses / Radiologic identification *Mass disaster *Practitioner self-protection	*Non-displaced rib fractures *Foreign bodies with attenuation similar to human tissue *Reconstruction of wound channels *Type of gas *Coronary thrombosis *Myocardial infarction *Pulmonary embolism *Sepsis *Intoxication	General recommendations	General recommendations	Radiation protection, infrastructure, preparation and transportation of the body, data archiving, personnel, teleradiology, demonstrative aids in court
Japan	2015	*Traumatic death *Drowning (caveats) *Carbonization (caveats) *Asphyxia(caveats) *Intoxication (caveats) *Hypothermic deaths (caveats) *Gas embolism (caveats) *Chronic physical abuse (caveats) *Natural (caveats)	*Myocardial infarction (caveats) *Pulmonary embolism (caveats)	N/A	N/A	Legal requirements for forensic imaging, article is specific to death occurring outside medical institutions, MRI
ESPR and ISFRI Guidelines	2019	* Written specifically for PMCT * Applications not specifically listed * Case-by-case in routine practice	N/A	Detailed acquisition protocol for children	Trained radiographer Qualified radiologist	Forensic imaging in children, international survey, routine practice, standardization for research
IAFR Guidelines	2020	*Not specific to PMCT *Sudden unexpected adult death * Sudden unexpected infant death *Road traffic accidents *Homicide *Suicide *Overdose *Deaths following medical intervention *Custodial death *Decomposed remains *Mass fatality *Sudden unexplained death in epilepsy	N/A	N/A	Specific for radiographers	Forensic imaging of the living, evidence, protocols, consent, confidentiality, medico-legal, records, involvement of students and assistant practitioners, reports, health and safety, mass fatality, employers
Royal College of Pathologists	2021	*Specifically, “cross-sectional imaging” Mentions: *Major trauma *Overdose *Natural death Recommends: *The decision for invasive autopsy should be made after postmortem imaging and external examination	Cannot be reliably diagnosed using nonenhanced cross-sectional imaging: *Sepsis (without abscess) *Toxic ad metabolic conditions *Primary inflammatory diseases *Pulmonary embolism *Intestinal ischemia	N/A	General recommendations	Audit, health and safety, PMCT angiography, image guided biopsy, toxicology
SIRM Italian	2021	*Major trauma *Identification *Gunshot injuries *Battered children *Drowning *Asphyxia *Carbonization *Gas embolism	*Soft tissues *Vascular lesions *Does note improved diagnostic power with PMCT angiography	*Protocols for adults and children	“Specialized radiologist”	Historical perspective, reporting, PMCT angiography, PMMRI
HPCSA	2023	*Not specific to PMCT *Investigation of non-fatal injuries *Foreign bodies *Human Identification *Determining Cause of Death for: *Road traffic deaths *Deaths following medical intervention *Homicide *Suicide *Custodial deaths *Decomposed human remains *Mass fatalities *Sudden infant deaths	N/A	N/A		Responsibilities of employer, responsibilities of radiographer, authentication, evidence, health and safety, records, confidentiality

### Natural deaths

Cardiovascular disease remains the most common cause of natural death in developed countries. Multiple imaging modalities are commonly employed for evaluation of cardiac pathology including radiography, PMCT, PMCTA, PMMR, and PMMRA. While radiography is the most accessible modality, it has significant limitations for basic forensic applications such as determining cardiac size and vascular calcification burden. Namely, magnification, poor soft tissue resolution, and superimposition. For instance, an enlarged cardiac silhouette on radiography has a primary differential diagnosis of cardiomegaly or pericardial effusion (diagnoses that are clearly evident on cross sectional imaging).

In fact, cross sectional imaging can provide supplementary evidence distinguishing simple pericardial effusions from hemopericardium [99, 100]. Furthermore, imaging characteristics can help distinguish antemortem versus postmortem pericardial haemorrhage and identify underlying pathology such as aortic dissection [101–103].

While there are conflicting studies regarding imaging as a complementary tool versus a standalone tool for assessment of cardiomegaly, cardiac size is commonly assessed by PMCT and PMMR [104–107]. On PMCT, Winklhofer et al. found that a cardiac thoracic ratio of 0.57 identified cardiomegaly with minimum specificity of 95%.

PMCT readily detects coronary artery calcifications, a key marker for general atherosclerotic burden of the body [108, 109]. Calcium scoring (Agatston’s score) is used clinically to assess cardiovascular risk of death and is often applied to postmortem imaging [110, 111]. In both contexts, it is important to acknowledge that a high Agatston score can be seen in individuals without cardiac events and that even a score of zero can be seen in individuals with cardiac events.

PMCTA goes beyond calcium scoring and can estimate luminal stenosis [103, 112–116]. In fact, Palmiere demonstrated that PMCTA precisely identified the type and location of acute and chronic coronary thrombosis [117]. PMCT and PMCTA can also identify myocardial rupture, and PMCTA may detect signs of myocardial infarction [112]. Thus, considering the risk of misinterpretation due to possible artefactual post mortem thrombi, pathological examination remains necessary.

While not widely accessible, PMMR is the premier imaging modality for myocardium. PMMR allows not only the visualization of myocardial infarctions but can also be used to date them using the variation of the signal of the necrotic centre and the marginal regions, in T1 and T2-weighted sequences [118–122]. In fact, Wagensveld et al. confirmed histologically that PMMMRI has a high sensitivity and specificity for the detection of acute and chronic myocardial infarctions [123].

More generally, post-mortem imaging is of particular interest in deaths of natural and non-criminal origin, as it can support the development and practice of minimally invasive autopsies, whose correlation rates with conventional autopsies are rather satisfactory [124]. If a conventional autopsy is not possible due to family refusal, post-mortem imaging (if possible with contrast injection), combined with targeted biopsies and samples, may prove a good compromise [112, 125]. These techniques should be employed in conjunction with, rather than as a replacement for, conventional autopsy in the context of forensic investigation or suspected criminal deaths.

### COVID-19 and pulmonary abnormalities

During the outbreaks of the COVID-19 pandemic, the diagnostic performance of CT scans in detecting lesions in living patients placed imaging at the forefront of the diagnosis and management of infected patients [126]. Rapidly, the application of CT, also in deceased people, has emerged and post-mortem imaging has been used in many health centres and forensic institutes with different purposes [127, 128]. First, it has been part of the global scientific movement for a better description and understanding of the disease [129, 130]. In addition to autopsies, it provided elements on the natural evolution of the infection and correlations with histological findings [131, 132]. The question of safety of the pathologists and forensic workers, facing a new and highly contagious infection, led to considering PMCT as a tool for screening decedents before performing autopsies, in addition to nasopharyngeal swabs [133]. Results showed that the most frequent radiological patterns in PMCT consist of ground glass opacities, generally associated with consolidation (mixed densities) and/or crazy paving, with a peripheral or diffuse distribution; traction bronchiectasis has also been described [132]. PMCT is considered relevant in providing evidence in favour of SARS-CoV-2 infection, particularly in the case of a suggestive clinical history. However, it should be interpreted with caution in the case of a potential asymptomatic infection, because there is considerable overlap between the findings in COVID pneumonia and other disparate pulmonary pathologies which can lead to false positives [131, 134]. Results of PMCT especially regarding the cause of death should systematically be associated with the complete post-mortem data to assess a SARS-CoV-2 infection.

Results of PMCT especially regarding the cause of death should systematically be associated with the complete post-mortem data to assess a SARS-CoV-2 infection.

Comprehensive knowledge of pulmonary anatomy (the forensic pathologists’ domain), pattern recognition, relevant clinical history (when known), and scene narrative details are crucial for accurate imaging assessments. While radiography was the original modality for pulmonary examination, PMCT is the industry standard.

The lungs are comprised of complex anatomy and are even more challenging to interpret in postmortem contexts compared to medical imaging. This is primarily related to the effects of hypostasis and decomposition, to which the pulmonary tissue is particularly susceptible [135–137]. Pulmonary hypostasis in forensic imaging is personified by dependent ground glass opacities, although care must be taken to account for the position of the body prior to scanning, as there may be anti-dependent and mixed dependency opacities [138, 139]. Postmortem changes in the lungs (similar to the rest of the body) include the development of fluid and gas collections. The postmortem changes may mask underlying pathology [140–142]. PMCT Ventilation, while not widely available, improves diagnostic quality by increasing aeration of the lungs while reducing the effects of livor [143–145].

Postmortem clotting renders identification of pulmonary embolism extremely challenging in forensic cases. It has been suggested that patterns of clot morphology can aid detection of pulmonary emboli on PMCT. Specifically, clots conforming to the shape of the vessel are described as more likely postmortem versus irregular clots, which are more likely antemortem [139, 146]. Correlation with femoral vein findings and inferior vena cava measurements have been discussed, although not found helpful [146, 147]. PMCTA examinations are more promising [103, 148–151]. However, PMCTA is still unable to differentiate thrombo-embolus from postmortem clots in many cases [39, 152, 153]. PMMR has shown promise, but is not typically available [104, 154–156]. In the future, perhaps image guided biopsy techniques will enhance minimally invasive detection of pulmonary embolism [157].

### Foetal and paediatric deaths

Exploring foetal, perinatal and infant death by post-mortem imaging is probably one of its most meaningful applications. Emotional charge following the loss of a child, including the loss of a pregnancy, is extremely high, leading to a steady decline in the acceptance of autopsies [158, 159]. Post-mortem imaging is an excellent tool to investigate the cause of death without altering the body, allowing radiologists, paediatricians, and pathologists to respect grieving parents’ wishes [160].

Post-mortem foetal imaging is best completed with a multimodality approach. It involves X-Ray, ultrasound, MRI (from 1,5T to 9,4T), computed tomography and micro-CT [161–163]. The purpose of postmortem imaging following a miscarriage, a termination of pregnancy or an in-utero foetal death is to provide a precise description of the morphological abnormalities [164, 165]. It will help to determine the cause of death and/or mechanism responsible for the disease or syndrome, in addition to genetics, biological samples and if accepted autopsy. PMMR is particularly relevant for detecting malformations and anatomical defects of the central nervous system or thoracic contents, but remains limited for diagnosing ischaemic diseases or infections [164, 166]. Postmortem imaging and especially PMMR is also the cornerstone of minimally invasive autopsies, with a better acceptance than conventional autopsy, and good diagnostic performance in comparison to conventional autopsy [167–169]. Post mortem ultrasound may also be helpful, with increased availability and the possibility to perform echo-guided biopsies [170–172].

Forensic applications of postmortem imaging in infant and children concern neonaticide (i.e. the death of a newborn in undefined circumstances), sudden unexpected death in infancy (SUDI), and violent and/or unexpected death in children and adolescents. It mainly rests on X-Ray, PMCT and PMMR [163, 173].

In neonaticide, postmortem imaging is a relevant tool to determine gestational age, assess findings that may indicate neonatal breathing by measurement of lung densities, and detect traumatic injuries [174, 175]. A series of cases has shown that measuring the density of lung parenchyma in CT scans using ROI can be used to determine the level of lung aeration, and therefore respiration, after birth, with an excellent correlation with microscopic analysis [175]. Caution remains necessary in cases of severe decomposition [176]. PMMR can be helpful to assess lung aeration and differentiate living versus stillbirth [177].

SUDI (i.e. the unexpected death of an apparently healthy child before the age of one year) was one of the first applications of PMCT [178, 179]. The aim of imaging in this context is before all to detect traumatic injuries, especially intracranial bleeding and bone fractures, as strong arguments for child abuse like abusive head trauma [180–183]. This is why it is essential that it is carried out in addition to an autopsy. It also provides first elements of the internal content of the body, that will guide the forensic pathologists to orientate biological and histological samples. Ideally, the full imaging work-up should include a skeletal survey (segment by segment, with anteroposterior and lateral views, particularly for the limbs), a whole-body PMCT and a PMMR, at least of the brain and spinal cord. A recent study clearly suggested that PMCT should not replace X ray for the detection of extremity fracture, as the sensitivity of PMCT is lower than skeletal survey for the detection of corner metaphyseal lesions, due to a lower spatial resolution [184]. Studies quite agree that the ability of postmortem imaging and especially PMCT to detect the cause of death in the case of SUDI is rather limited in comparison to autopsy [180, 185, 186]. In contradistinction to foetal death, SUDI is rarely linked to a congenital malformation that could be easily and with certainty diagnosed by imaging. Natural causes of death like infections, aspiration, heart defects or metabolic disease remain mostly unseen by post-mortem imaging [187].

However, PMCT seems to have a good diagnostic accuracy for detecting traumatic injuries especially in older children [188]. It is to be noted that there are currently few studies exploring the added value of postmortem imaging and traumatic injury patterns visible in children aged between 2 and 18, deceased by violent death [186]. Exploring the specificity and particularities of traumatic injuries in children by post mortem imaging in cases of traumatic death could be a relevant area of research in the years to come.

### Strangulation and hanging

PMCT and MRI have been studied for evaluation of strangulation and hanging. In this context, multiple studies reported that PMCT has the advantage of identifying skeletal injuries that are challenging to detect at autopsy [189–191]. On the contrary, Graziani et al. found a clear advantage for conventional autopsy in the identification of hyoid and laryngeal fractures in the setting of strangulation, however, advised the use of both PMCT and conventional autopsy given his finding of low overall agreement between both practices [192]. A systematic literature review by Gascho et al. found that PMCT’s sensitivity for hyoid and thyroid fractures is equal to that of conventional autopsy [193]. PMCT is also not sensitive for the detection of regional haemorrhage [193, 194]. Similar to medical imaging, PMMR has been reported to improve the depiction of haemorrhage and other regional soft tissue findings [189, 195–199].

One advantage of imaging is the ability to detect gas collections [200, 201]. Gas within the soft tissues of the neck has been reported as a “vital” sign in strangulation and hanging deaths [193, 202–204]. Imaging may also reveal supportive evidence in other organs, such as pulmonary oedema, aspiration, gastric contents suggestive of pills, and cerebral oedema [193, 201, 205, 206]. In uncomplicated hangings, whole body imaging, most frequently performed by PMCT, can exclude other internal injuries that would conflict with the assumption of suicide [140, 190, 207].

## Other applications

### Anthropology and archaeology

The basic use of radiology was first to transpose staging and techniques used in forensic anthropology.

Then, specific radiological methods, new classifications and staging were described and tested.

Age assessment in legal procedures can be done with radiographies completed by CT of the clavicle when the wrist bone maturation is achieved [208, 209]. In this application, CT of the clavicle was a real “game-changer,” enabling clear and understandable recommendations and stagings [210]. Furthermore, new classifications and works now include statistical information and data which are very important for assessing the scientific quality of research and results. Current research however indicates that MRI staging is possible and accurate, which is a good point, as it could replace the radiation-based imaging methods [211]. Even if forensic anthropology is an old and respectable speciality, with already an extensive description of the various ways of determining or assessing different anthropological parameters on a wide variety of bones, teeth., virtual anthropology is still a young speciality! Consequently, new scientific works dealing with sex determination and age assessment are being published [212, 213].

The democratization and generalization of access to CT platforms opens up new possibilities for archaeologists: micro-excavation of cremation urns, for example. Making a CT before micro-excavating the urn is also very useful and allows the archaeologist to anticipate what he or she will find in the urn: bones, stones, metal fragments, etc [214]. The introduction of morphometric geometric analysis is also important to estimate sex differences by differentiating size and shape related differences [215–217]. This can be done on pelvic bones, orbital bones, skull, face, etc., quantifying the dimorphic differences in terms of shape differences [218]. It may also be used to quantify secular changes [219].

Technical progress concerning post processing data has led to new visualizations of the bones. A newly introduced technique, global illumination rendering, showing more photorealistic 3D images than those achieved with the standard volume rendering technique (VRT), and could be perfectly applied to methods using morphological criteria for age estimation. For example, global illumination rendering was used for age estimation based on acetabulum analysis [220]. Last, many possibilities and promising results are also coming concerning age estimation and sex determination using artificial intelligence and machine learning.

### Animal models - experimental research

Forensic imaging research is mainly clinical research, seeking to investigate the roles and benefits of post-mortem imaging in everyday practice. There are, however, a few experimental studies, notably using animal models, that need to be noticed. In opposite to human cadavers, animal models provide the possibility of reproducibility on a higher number of subjects. In forensic research, the most common species used as animal models are rats, pigs, mice and rabbits, on various themes such as trauma analysis, post-mortem interval and taphonomy, toxicology or wound aging [221]. The rare studies describing postmortem imaging research on animal models used piglets or sheep (sometimes only legs) for the study of ballistic trauma or non-accidental fractures [222–225]. Hyodoh et al. tried to precisely characterize lung abnormalities in PMCT following different manner of death (i.e. drowning, hypothermia, suffocation) and different post mortem intervals in rabbits [226]. The advantages of medium-sized animals are their accessibility and ease of care, in comparison to other species such as cows. These characteristics make them good candidates for imaging studies, unlike small animals like rats, whose small size would be too limiting a factor for the spatial resolution of most machines [222]. In 2019, Mole et Heyns raised the ethical issues surrounding the use of animal models in forensic science research: they suggested that imaging could be an alternative to euthanasia and autopsy in certain specific cases [221].

Other experimental research with a forensic application could be cited: the use of dual-energy CT scans to determine the nature of the metal (lead vs. copper) in bullets, or to analyse the behaviour of heroin, cocaine and their adulterants [225, 227, 228].

## Conclusion

Today, post-mortem examination cannot be considered without integrating post-mortem imaging. From paediatric and perinatal death to charred bodies, post-mortem imaging provides crucial elements to bring light on the cause and the circumstances of death. Its accuracy is particularly important for detecting metallic foreign bodies and revealing bone fractures, especially in anatomic areas that are difficult to explore by autopsy. Advances in scientific research over the last decades have clarified the benefits and limitations of post-mortem imaging, guiding forensic pathologists all over the world to adapt their practice and use imaging appropriately.

## Data Availability

Non-applicable (review).

## Code availability

Non-applicable (review).
